# Retraction Note: Accuracy of Genomic prediction for fleece traits in Inner Mongolia Cashmere goats

**DOI:** 10.1186/s12864-025-11353-y

**Published:** 2025-02-19

**Authors:** Xiaochun Yan, Jinquan Li, Libing He, Oljibilig Chen, Na Wang, Shuai Wang, Xiuyan Wang, Zhiying Wang, Rui Su

**Affiliations:** 1 College of Animal Science, Inner Mongolia Agricultural University, Hohhot, Inner Mongolia Autonomous Region 010018 China; 2 Inner Mongolia Key Laboratory of Sheep & Goat Genetics Breeding and Reproduction, Hohhot, Inner Mongolia Autonomous Region 010018 China; 3 Key Laboratory Of Mutton Sheep & Goat Genetics And Breeding, Ministry of Agriculture And Rural Affairs, Hohhot, Inner Mongolia Autonomous Region 010018 China; 4 Engineering Research Centre for Goat Genetics and Breeding, Inner Mongolia Autonomous Region, Hohhot, Inner Mongolia Autonomous Region 010018 China; 5 Inner Mongolia Jinlai Livestock Technology Co., Ltd, Hohhot, Inner Mongolia Autonomous Region 010018 China; 6 Inner Mongolia Yiwei White Cashmere Goat Co., Ltd, gion, Ordos, Inner Mongolia Autonomous Re 010018 China; 7 Livestock Improvement Center of Alxa Left Banner, Alxa League, Inner Mongolia Autonomous Region 75000 China

**Retraction Note: BMC Genomics 25, 349 (2024)**

**https://doi.org/10.1186/s12864-024-10249-7**

The Editor has retracted this article because it contains material previously published in two articles with common authors. Specifically, the genotype data, ssGBLUP analysis methods and results, and a significant portion of the data in Table 1 were previously published in [1], and Figure 2 was previously published in [2].

Xiaochun Yan did not explicitly state whether they agree or disagree with this retraction. The remaining authors did not respond to correspondence from the Publisher regarding this retraction.
